# Modulation of neuromuscular excitability in response to acute noxious heat exposure has no additional effects on central and peripheral fatigability

**DOI:** 10.3389/fphys.2022.936885

**Published:** 2022-08-12

**Authors:** Nerijus Eimantas, Soneta Ivanove, Neringa Baranauskiene, Rima Solianik, Marius Brazaitis

**Affiliations:** Institute of Sport Science and Innovations, Lithuanian Sports University, Kaunas, Lithuania

**Keywords:** motor drive, fatigue, hyperthermia, thermal therapy, thermal afferents

## Abstract

**Background:** Whole-body hyperthermia (WBH) has an adverse effect on the nervous system and neurophysiological performance. In the present study, we examined whether short-duration whole-body immersion in 45°C water (HWI-45°C), which produces a strong neural and temperature flux without inducing WBH, can increase or impair neurophysiological performance in humans.

**Methods:** Fifteen men (aged 25 ± 6 years) were enrolled in this study and participated in three experiments: 1) a brief (5-min) immersion of the whole body in 37°C water (WI-37°C); 2) a brief (5-min) HWI-45°C; and 3) a control trial in a thermoneutral condition at an ambient temperature of 24°C and 60% relative humidity. Before and after the immersions, neuromuscular function (electromyographic activity, reflexes, electrically and voluntary induced torque production, voluntary muscle activation level) were tested. To provoke central inhibition, the participants performed a sustained 2-min maximal voluntary contraction (MVC).

**Results:** Thermophysiological strain was greater after HWI-45°C than after WI-37°C. Electrophysiological modulations of motor drive transmission and peripheral modulations of muscle contractility properties in response to HWI-45°C seemed to have little effect on central activation of the exercising muscles and no effect on MVC production.

**Conclusion:** Although exposure to acute noxious heat was effective in evoking neuromuscular excitability, the increases in core temperature (∼0.2°C) and muscle temperature (∼0.6°C) did not induce moderate or severe WBH. These changes did not seem to affect central structures; that is, there were no additional increases in central and/or peripheral fatigue during a sustained 2-min MVC.

## Introduction

Sudden application of external noxious hot (>42°C) stimuli to the body skin surface, for example hot water immersion (HWI), triggers high-temperature-sensitive receptors (TRP) by causing a rapid volley of impulses in cutaneous nerve endings and by sensitizing the preoptic area and insula to increase arousal and alertness as part of the thermoregulatory and sensation responses (Patapoutian et al., 2003; Tóth et al., 2014; Hoffstaetter et al., 2018). Recent studies in knockout mice have identified a TR*i*Plet of ion channels that involve overlapping expression of the TRP vanilloid 1, TRP ankyrin 1, and TRP melastatin 3 channels, which mediate the current and cause a maximal noxious heat-sensing response (Vandewauw et al., 2018; Vriens and Voets, 2019). From the evolutionary view, overlapping expression of these thermo-TRP channels in nociceptor neurons with a current activity threshold of >42°C has been suggested to represent a powerful mechanism that ensures avoidance of noxious hot temperatures (Vandewauw et al., 2018; Vriens and Voets, 2019).

In humans, the strong cutaneous neural flux during the first minute of whole-body immersion in 45°C (vs. ≤41°C) water has been shown to evoke an acute cardiorespiratory shock response (Eimantas et al., 2022). Importantly, short-duration (5-min) noxious HWI at 45°C leads to a mild increase in rectal (∼0.2°C), deep muscle (∼0.6°C at a depth of 3 cm), and skin (∼7.1°C) temperatures and is sufficient for potentiating the thermoregulatory increase in heart rate (∼20 bpm), sweating, and perceptual and respiratory strain responses (Eimantas et al., 2022). Whole-body hyperthermia (WBH), defined as an increase in body core temperature >38.5°C, has an adverse effect on the nervous system, as evidenced by synaptic failure, perturbation of supraspinal neural drive generation, increased tension at the voltage-gated sodium channels of a single axon potential, and neurophysiological performance (Bolton et al., 1981; Caputa et al., 1986; Rutkove et al., 1997; Fuller et al., 1998; Karunanithi et al., 1999; Kelty et al., 2002; Todd et al., 2005; Racinais et al., 2008; Brazaitis et al., 2012, 2015; Périard et al., 2014). However, it is unknown whether short-duration whole-body immersion in noxious 45°C water, which produces a strong neural and temperature flux without inducing the WBH, improves or impairs neurophysiological performance in humans.

The main purpose of our study was to determine whether short-duration (5-min) whole-body noxious HWI that does not induce WBH affects central changes, as measured by the central activation ratio (CAR), mean frequency (MnF), and root mean square (RMS) of the surface electromyography (sEMG) signal, H-reflex, and V-wave, and peripheral changes including changes in contractile properties of the calf muscles and M-wave in young healthy men. Given that the temperature-dependent modulation correlates partly with the presynaptic inhibition or activation mediated by temperature-sensitive group III and IV afferents (Bigland-Ritchie et al., 1986; Duchateau et al., 2002; Avela et al., 2006)**,** we hypothesized that spinal modulation of neural drive would be accelerated, as shown by decreases in the amplitude and latency time of evoked H-reflex and V-waves by acute short-duration (5-min) whole-body immersion in 45°C (vs. 37°C) water. Based on previous studies, we also expected that superficially potentiated heat fluxes in deep muscle tissues and the body core caused by HWI-induced peripheral vasodilation (Bonde-Petersen et al., 1992; Brazaitis et al., 2017) would increase the contraction and half-relaxation speeds, as measured by a shorter contraction time (CT) and shorter half-relaxation time (HRT), respectively, of the calf muscle. If so, one would expect that the decreased amplitude and increased conduction velocity of the reflex responses as well as accelerated muscle contractility induced by HWI in normothermic people would require greater energetic resources (faster CT + HRT, increased MnF, and decreased RMS) for a sustained 2-min maximal voluntary contraction (MVC) by providing greater inhibitory feedback to central structures, which may contribute to greater central fatigue (i.e., supraspinal failure) (Racinais et al., 2008).

## Materials and methods

This work is a follow-up to a recently published study (Eimantas et al., 2022). The information related to the experimental design, the body temperature measurement, and whole-body immersion procedure has already been described in the previous article. In this latter study, we examined whether short-duration whole-body immersion (for 5 min) in noxious hot water (45°C) is a sufficient stimulus to induce a respiratory acute shock response.

### Participants

Eighteen male volunteers were assessed for eligibility. Participants were excluded if they smoked or had Raynaud’s syndrome, asthma, a neurological pathology, or another condition that could be worsened by acute exposure to hot (45°C) water. The inclusion criteria were as follows: 1) aged 20–30 years; 2) no excessive regular sport activities (i.e., <3 times per week and <150 min of moderate intensity or <75 min of vigorous intensity activity per week); 3) no involvement in any temperature-manipulation program or extreme temperature exposure for 3 months; 4) no medications or dietary supplements that could affect experimental variables; 5) no needle phobia; 6) sufficient tolerance to electrical stimulation; and 7) regular sleep schedule (7–9 h of sleep per night). Fifteen men met the inclusion criteria and agreed to participate in this study. The physical characteristics of the participants are presented in Table 1.

**TABLE 1:** Physical characteristics of the participants in the study.

Number of participants	15
Age, yr	25 ± 6
Height, cm	187.40 ± 6.17
Mass, kg	88.69 ± 7.77
Body mass index, kg m^−2^	25.28 ± 2.19
Body fat, %	17.93 ± 4.40
Mean skinfold thickness, mm	11.85 ± 5.41
Body surface area, m^2^	2.15 ± 0.61

Values are expressed as the mean ± standard deviation.

Written informed consent was obtained from all participants after explanation of all details of the experimental procedures and the associated discomforts and risks. All procedures were approved by the Human Research Ethics Committee (No. BE-2–30) and were conducted according to the guidelines of the Declaration of Helsinki, with the exception of registration in a clinical trials database. The participants were in self-reported good health, which was confirmed by a medical history and physical examination.

## Experimental procedures

### Familiarization session

About 2–3 days before the experimental trial, each participant attended a familiarization session. Upon arrival at the laboratory, anthropometric variables and body composition were measured, and the experimental procedures used for neuromuscular testing were demonstrated. The tolerance to electrical stimulation was assessed on relaxed muscle by stimulating the tibial nerve with a 250-ms test train of stimulation at 100 Hz (TT100). The volunteers learned to achieve a maximal isometric effort of ankle plantar flexion and to maintain it for 3–4 s. They were instructed to refrain from consuming any food for at least 12 h, ingesting alcohol and caffeine, and engaging in heavy exercise for at least 24 h, and to sleep at least 7 h before each experimental session. To standardize the state of hydration and the feeling of thirst, the participants were allowed to drink still water without any restriction. On arrival to the laboratory, all participants voided their bladders to estimate the level of hydration based on the specific gravity of urine (PocketChem UA PU-4010, Arkray Factory Inc., Kyoto, Japan) and all men were found to be well hydrated (SG, 0.010–0.020) prior all experimental trials. Tests were performed at an ambient temperature of 24°C and 60% relative humidity.

### Experimental protocol

Studies suggests that passive heating allowing to obtain the increase in muscle or core temperature achieved by active warm up without depleting energy substrates may improve short-term and intermediate physical performance (∼10 s–5 min) (Bishop, 2003). Our recent study established that 5-min HWI at 45°C results in an increase in T_re_ by 0.21 C and T_mu_ by 0.59°C (Eimantas et al., 2022). Thus, the study consisted of experiments involving 1) a brief (5-min) immersion of the whole body in 37°C water (WI-37°C trial); 2) a brief (5-min) immersion of the whole body in 45°C water (HWI-45°C trial); and 3) a control trial in a thermoneutral condition at an ambient temperature of 24°C and 60% relative humidity (CON trial). These trials were performed in a balanced random order (crossover design) at least 1 week apart. We used the IBM Statistical Package for the Social Sciences (SPSS) for Windows (version 22.0; IBM Corp., Armonk, NY, United States) to determine the order of tests for each participant.

### CON trial

**FIGURE 1:**
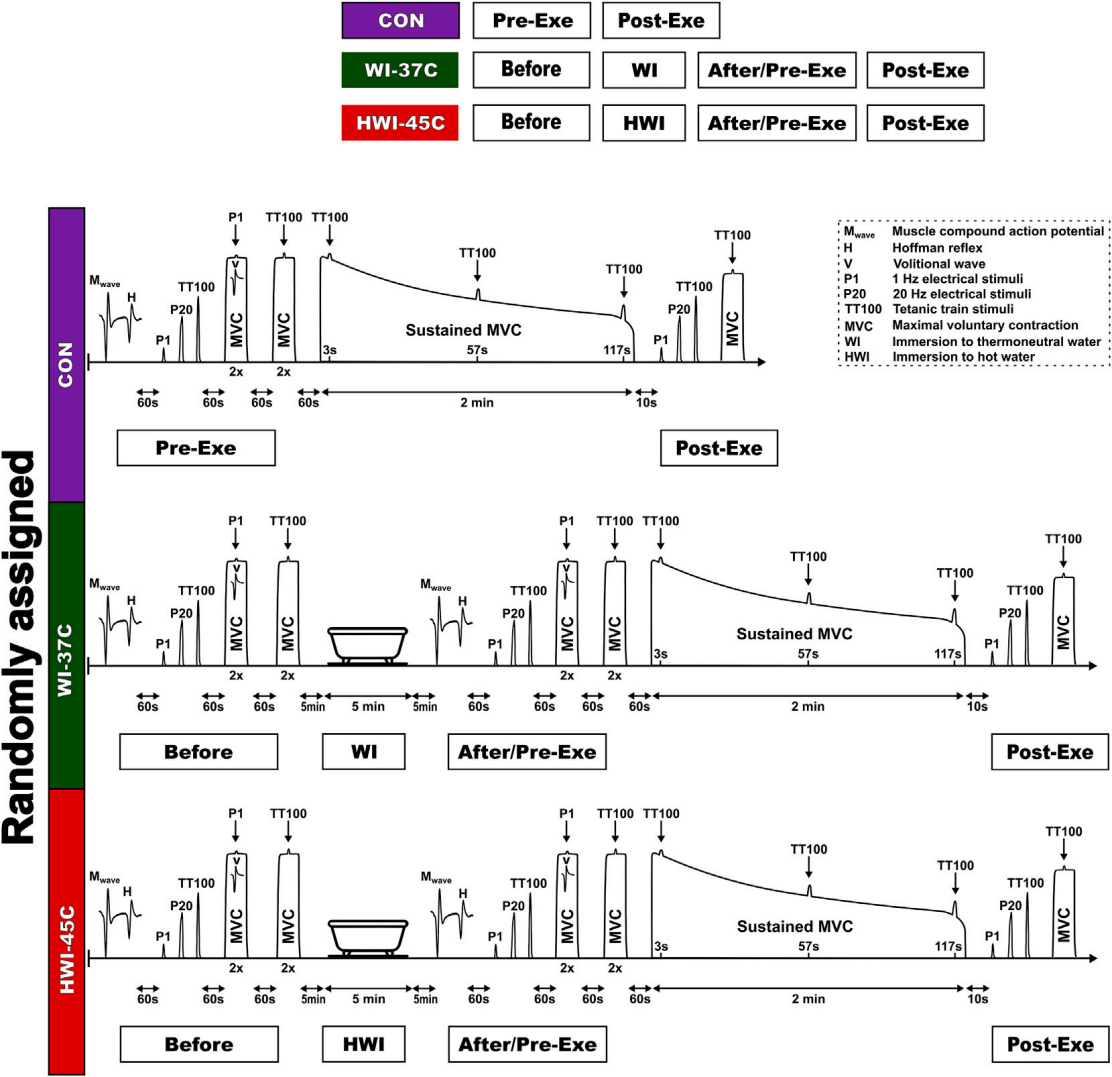
Schematic representation of the experimental design.

On arrival at the laboratory, the participant rested in semirecumbent position for 20 min while dressed in a T-shirt, swim shorts, and socks. Resting heart rate (HR), skin temperature (T_sk_), muscle temperature (T_mu_), and rectal temperature (T_re_) were recorded. Within ∼5 min after these resting measurements, the participant was seated in the dynamometer chair, stimulating electrodes were placed over the tibial nerve, sEMG electrodes were placed over the soleus (SOL) muscle of the right leg, and reflexes were assessed (Figure 1) (see “*Reflex recordings*”). After a 1-min rest, the force-generating capacity of the posterior calf muscle was assessed by applying single-twitch (P1) nerve stimulation and 1-s trains of electrical stimuli at P20 and TT100 (see “*Torque-generating capacity measurement*”). Each stimulus was delivered automatically to the relaxed muscle with a 5-s rest interval between each.

To determine the supraspinal excitability (V_sup_) response, two attempts were performed at an MVC of 3–4°s, with a 1-min rest interval between each, by applying P1 stimulus superimposed on the voluntary contraction. Next, to determine the CAR of the exercising muscle, two attempts were performed at an MVC of 3–4 s, with a 1-min rest interval between each, by applying a TT100 stimulus superimposed on the voluntary contraction. After a 1-min rest, a 2-min isometric MVC was then performed. The TT100 was superimposed on the contraction at about 3, 57, and 117 s. The measurement at 10 s was repeated after the end of the 2-min MVC to measure involuntary torque-generating capacity by imposing P1 and 1-s trains of electrical stimuli at P20 and TT100, and MVC torque and CAR were measured.

### WI-37°C and HWI-45°C trials

Baseline body temperatures, reflex recordings, torque-generating capacity, MVC, and CAR were measured as described for the CON trial. After neuromuscular testing, the participant began a short-duration (5-min) head-out immersion in innocuous warm (37°C) water (WI-37°C trial) or noxious hot (45°C) water (HWI-45°C trial) (Figure 1). During immersion, the participant remained in a semirecumbent posture with his arms folded across the chest and the legs extended almost straight and together. Within ∼1 min after leaving the bath, the volunteer was towel dried and body temperatures were measured. After the end of the water immersion procedure, neuromuscular testing was performed in the same order as before the water immersion. Thereafter, the 2-min isometric MVC was assessed and then involuntary torque-generating capacity, MVC, and CAR were assessed as described above for the CON trial.

## Experimental measurements

### Anthropometric and body composition measurements

Body mass and body fat were measured using a body composition analyzer (TBF-300, Tanita, Arlington Heights, IL, United States), and the body mass index was calculated. Body surface area (BSA) was estimated according to the formula: BSA = 128.1 × weight^0.44^ × height^0.60^ (Tikuisis et al., 2001). Skinfold thickness was calculated as the average thickness of 10 skinfold sites (chin, subscapular, chest, side, suprailium, abdomen, triceps, thigh, knee, and calf) (McArdle et al., 1984) using a medical skinfold caliper (SH5020, Saehan, Masan, and South Korea).

### Body temperature measurements

T_mu_ was measured before all trials and within 1 min of leaving the bath in the WI-37°C and HWI-45°C trials. T_re_ and T_sk_ were measured before all trials and immediately and 15, 30, and 60 min after leaving the bath or at the same time points in the CON trial. T_re_ was measured using a thermocouple (accuracy, ± 0.01°C, Rectal Probe, Ellab, Hvidovre, Denmark) inserted to a depth of 12 cm past the anal sphincter. The rectal thermistor sensor was placed by each participant (Pranskunas et al., 2015). T_sk_ was measured with thermistors (accuracy, ± 0.01°C, Skin/Surface Probe, DM852, Ellab) at three sites: midline of the anterior surface of the right scapula (back), anterior surface of the right thigh (thigh), and midline of the posterior surface of the right forearm (forearm). The mean T_sk_ was calculated using the equation T_sk_ = 0.5_back_ + 0.36_thigh_ + 0.14_forearm_ (Burton, 1935). T_mu_ was measured with a needle microprobe (accuracy, ± 0.01°C, Intramuscular Probe, MKA, Ellab) inserted to a depth of 3.5 cm under the skin covering the largest bulk of the lateral gastrocnemius muscle in the right leg. For skin preparation before each T_mu_ measurement, the skin was shaved and disinfected before and after insertion of the microprobe using a cotton wool pad soaked with medicinal alcohol. No local anesthesia was administered before insertion. After the first measurement, the insertion area was marked with a circle with a diameter of 0.5 cm to ensure that the same insertion point was used in later measurements (Brazaitis et al., 2019).

### HR measurement

HR was measured before and throughout the CON, WI-37°C, and HWI-45°C trials using an HR monitor (V800, Polar Electro OY, Kempele, Finland).

### Physiological strain index

The physiological strain index (PSI) was calculated using the following equation of Moran et al. (1998):

$$PSI=5\left(T_{ret}-T_{re0}\right)\times {\left(39.5-T_{re0}\right)}^{-1}+5\left(HR_{t}-HR_{0}\right)\times {\left(180-HR_{0}\right)}^{-1}$$

The measurements for PSI were taken before (T_re0_ and HR_0_) and at the end of passive heating (T_re*t*_ and HR_*t*_). T_re_ and HR were assigned the same weight by using a constant of 5. Thus, the index was scaled to a range of 0–10, with 1–2 indicating no/little heat stress; 3–4, low heat stress; 5–6, moderate heat stress; 7–8, high heat stress; and 9–10, very high heat stress. The limits of the following values were used: 36.5°C ≤ T_re_ ≤ 39.5°C and 60 ≤ HR ≤ 180 bpm.

### Torque-generating capacity measurement

The isometric torque of the ankle plantar flexion muscles was assessed using an isokinetic dynamometer (System 4; Biodex Medical Systems, Shirley, NY, United States) calibrated according to the manufacturer’s service manual with a correction for gravity using the Biodex Advantage program (version 4. X). The participant was seated in the dynamometer chair with the trunk inclined at 70° with respect to the vertical and with hip, knee, and ankle joint angulations of 90°, 160° (full knee extension, 180°), and 90°, respectively. The foot was strapped securely to a dynamometric pedal. Hard cushioning (1 level) setting was used to obtain more reliable measurements (Ling et al., 1999).

Stimulating electrodes were fixed over the tibial nerve in the popliteal space, and sEMG electrodes were placed over the SOL muscle of the right leg. The positions of the electrodes were marked.

To measure the brief isometric MVC torque (Nm), the participant was asked to achieve and maintain maximal effort of ankle plantar flexion for 3–4 s. The best value of all attempts was used in subsequent analyses. To assess 2-min sustained MVC endurance, the participant was instructed to achieve and maintain a maximum effort of ankle plantar flexion for 120 s. During the 2-min MVC, the TT100 stimulus was superimposed on the contraction at 3 s (MVC-3), 57 s (MVC-57), and 117 s (MVC-117) to assess the CAR of the plantar flexors (Brazaitis et al., 2015; Solianik et al., 2015)**.** The CAR was calculated using the following equation: CAR = MVC/(MVC + TT100), where a CAR of 1 indicates complete activation, and a CAR <1 indicates central activation failure or inhibition.

During the MVC, each trace was examined visually to ensure that there were no artefactual spikes at the start of the signal curve. The participant’s arms were crossed on his chest and his hands grasped the trunk-supporting belt during all tests on the dynamometer. To ensure a maximal effort, standardized verbal encouragement was provided by the same experienced researcher during each voluntary ankle plantar flexion.

The positioning of the participant during the electrical stimulation assessment was essentially the same as that described above. A high-voltage stimulator (Digitimer DS7A, Digitimer, Hertfordshire, United Kingdom) was used to deliver 0.5-ms square-wave pulses at a constant current of 100 mA and constant voltage of 200 V (Paulauskas et al., 2020). Peak torques induced by electrical stimulation at 1 Hz (P1; representing the properties of muscle excitation–contraction coupling) and at 20 Hz (P20; representing the steep section of the force–frequency relationship curve) were measured. The peak torque amplitude (Nm; measured from the baseline to the peak torque) and the contraction plus half-relaxation time (CT + HRT) were measured for the resting TT100 contractions. The CT + HRT was calculated as the time taken for the torque to increase to the peak value and then to decrease to half of that value. Electrical stimulations were delivered to the resting muscle with a 3-s rest interval between each.

### Peak rate of torque development

The contractile peak rate of torque development (RTD) was determined from the electrically induced (TT100-RTD) isometric moment of torque. The peak RTD was determined as the peak slope of torque per 10 ms (Δtorque/Δ10 ms) (Paulauskas et al., 2020).

### Reflex recordings

SOL muscle H-reflexes, V-waves, and M-waves were evoked by 0.5-ms square-wave pulses stimulated by a cathode placed in the popliteal cavity and an anode placed over the posterior tibial nerve distal to the patella, with an interelectrode distance of ∼4 cm (Paulauskas et al., 2020). The resting maximum H-reflex (H_max_), which reflects the efficiency of transmission in Ia afferent motor neuron synapses, and maximum M-wave (M_max_), which reflects sarcolemmal excitability, were obtained by increasing the electrical intensity by 1 mA every 10 s over a 10–100-mA range at a constant voltage of 200 V. With increasing stimulation intensity, the H-reflex response initially increased progressively and then decreased and disappeared, whereas the M-wave achieved a maximum value and remained stable thereafter. Subsequently, the participant was instructed to perform two brief MVCs of the plantar flexor muscles for 3–4 s, with a rest period of at least 1 min between contractions. A superimposed stimulus (at M_max_ intensity) was evoked to obtain the V-wave (V_sup_). The peak-to-peak amplitude of the V-wave reflects the magnitude of the central descending neural drive to spinal motor neurons, although spinal factors such as motor neuron excitability and pre- or postsynaptic inhibition may also be involved (Aagaard et al., 2002). The M-wave amplitude was also used to normalize the amplitude of the recorded reflex waves (i.e., H_max_/M_max_ ratio), to ensure that any changes in the evoked H_max_ amplitude and latency reflected changes that occurred at the muscle fiber membrane or neuromuscular junction. The latencies of the electrically evoked action potentials were calculated from the stimulation artifact at the peak of the wave.

### Muscle activity-generating capacity

The skin was prepared carefully via shaving, abrasion, and cleaning with alcohol. Bipolar Ag–AgCl surface bar electrodes (diameter, 10 mm; center-to-center distance, 20 mm) (DataLog type no. P3X8 USB; Biometrics, Ltd., Gwent, UK) were placed over the SOL muscle at two-thirds of the distance between the medial condyle of the femur and the medial malleolus. The electrode position was marked with a waterproof pen, and the participants were asked to keep the mark throughout the experiment to ensure that the same recording site was used in all experimental trials. The ground electrode was positioned on the ankle of the resting leg. The sEMG signals were recorded by an amplifier with a gain of 1,000 and signal measurements using a third-order filter bandwidth of 20–460 Hz. The analog signal was sampled and converted to digital form at a sampling frequency of 5 kHz (Daniuseviciute et al., 2013). The sEMG analysis was performed by calculating the RMS as a measure of the sEMG amplitude values for a 1000-ms epoch coinciding with a 1-s force interval just before each P1 stimulus was superimposed on an MVC. The frequency content of an sEMG signal was assessed as the MnF.

### Statistical analysis

The number of participants was selected based on the calculated sample effect size after analyzing the data for the first five participants. At an α value of 0.05 and β (power) value of 80%, our power analysis indicated that, in a within-condition comparison, 15 participants would be required to detect a large effect (*p* < 0.05; *η*_*p*_^*2*^ > 0.25) for the hypothesized parameters.

The data were tested for normality using the Shapiro–Wilk test before parametric statistical analyses were performed, and all data were found to be normally distributed. Differences between the three trials (CON vs. WI-37°C vs. HWI-45°C) for the baseline body temperatures were analyzed using one-way repeated-measures analysis of variance (ANOVA) and Tukey’s adjustment for within-subject factors.

Two-way repeated-measures ANOVA was used to analyze: 1) the effects of the three temperature conditions (CON, WI-37°C, and HWI-45°C) and time (before vs. immediately, 15, 30, and 60 min after) on changes in T_re_ and T_sk_; 2) the effects of the two temperature conditions (WI-37°C vs. HWI-45°C) and time (before vs. after) on the changes in T_mu_, HR, MVC, CAR, spinal, and supraspinal reflex excitability, electrically induced muscle properties (P1, P20, TT100, CT + HRT, and TT100-RTD), and RMS and MnF of the SOL sEMG signal; 3) the effects of the three temperature conditions (CON, WI-37°C, and HWI-45°C) and sustained exercise time (before vs. MVC-3, MVC-57, MVC-117, and MVC-130) on MVC torque, CAR, RMS, and MnF of the SOL sEMG; and 4) the effects of the three temperature conditions (CON, WI-37°C, and HWI-45°C) and time (before and after exercise) on the percentage change in electrically induced muscle properties. When significant main effects were found, Sidak’s *post hoc* adjustment was used for multiple comparisons across a set of conditions within each repeated-measures ANOVA. A dependent-sample *t* test was used to locate differences in time and condition. The partial eta squared (*η*_*p*_^*2*^) was estimated as a measure of the effect sizes for temperature and time. Significance was defined as *p* < 0.05. Descriptive data are presented as mean ± standard deviation (SD). Statistical analyses were performed using IBM SPSS Statistics (v. 22; IBM Corp., Armonk, NY, United States).

## Results

### Body temperatures, HR, and PSI

**FIGURE 2:**
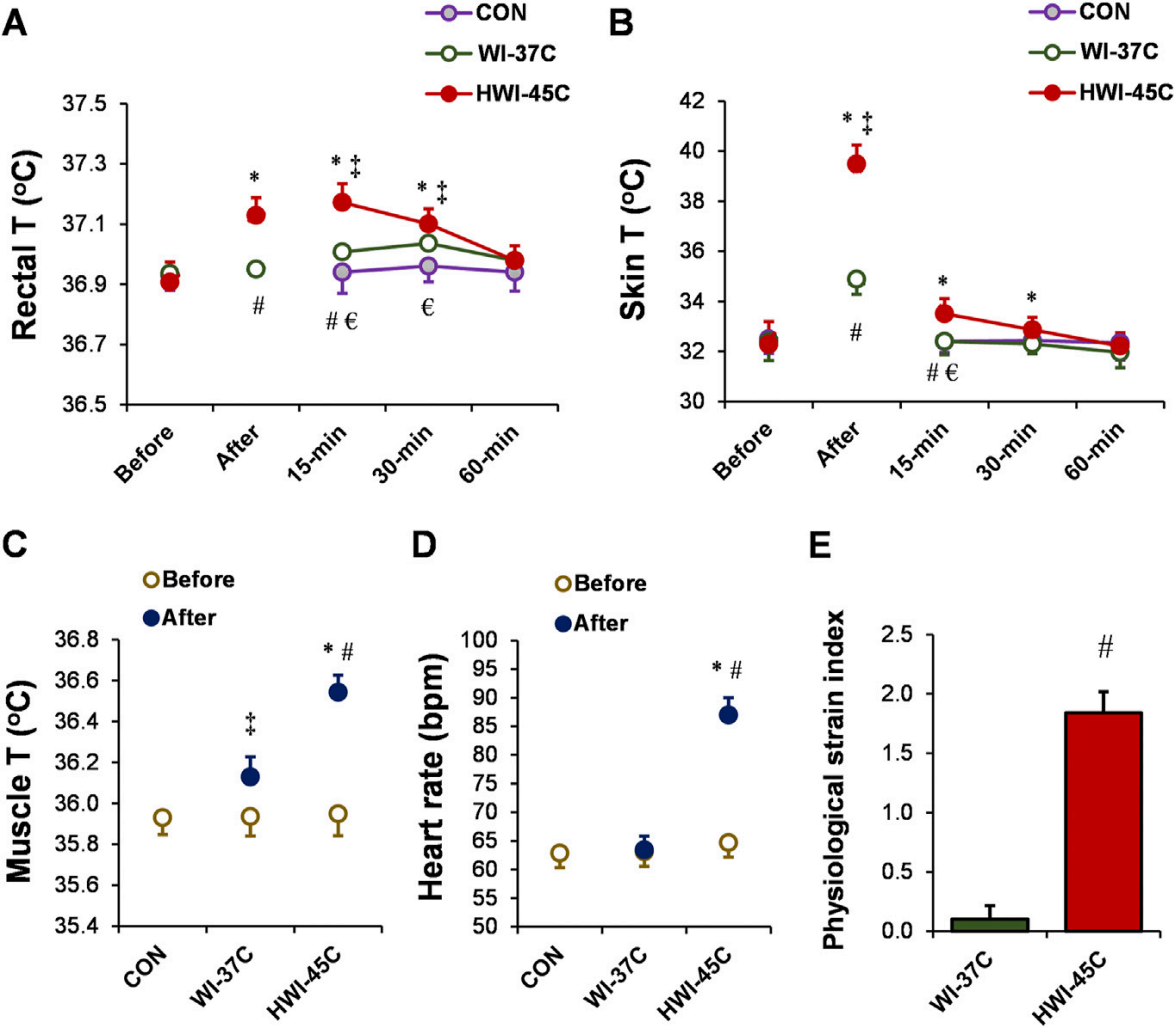
Changes in the rectal **(A)**, skin **(B)**, and muscle **(C)** temperatures (T), and heart rate **(D)** during whole-body immersion in water temperatures of 37°C (WI-37°C) and 45°C (HWI-45°C), and the control (CON) condition under a thermoneutral environment. The physiological strain index **(E)** in WI-37°C and HWI-45°C. *p < 0.05, HWI-45°C compared with before; ‡*p* < 0.05, WI-37°C compared with before; #*p* < 0.05, HWI-45°C compared with WI-37°C; € *p* < 0.05, HWI-45°C compared with CON. Values are expressed as mean and SD.

Baseline T_re_ (Figure 2A), T_sk_ (Figure 2B), T_mu_ (Figure 2C), and HR (Figure 2D) were similar across all trials (*p* > 0.05; *η*_*p*_^*2*^ < 0.1). Body temperature and HR remained unchanged throughout the 60-min experimental period in the CON condition (*p* > 0.05; *η*_*p*_^*2*^ < 0.1). By contrast, T_re_, T_sk_, and T_mu_ increased in both temperature conditions (WI-37°C and HWI-45°C) (time effect: *p* < 0.01; *η*_*p*_^*2*^ < 0.38). The increases in T_re_, T_sk_, and T_mu_ were significantly greater in HWI-45°C than in WI-37°C (trial effect: *p* < 0.01; *η*_*p*_^*2*^ < 0.29), and the time × trial interaction was also significant (*p* < 0.01; *η*_*p*_^*2*^ > 0.25). More detailed analyses revealed that T_re_ and T_sk_ increased immediately after immersion in HWI-45°C and remained elevated for 30 min after immersion (compared with before: *p* < 0.01; *η*_*p*_^*2*^ > 0.34). By contrast, in WI-37°C, T_re_ showed a delayed increase from 15 to 30 min after immersion, and T_sk_ increased immediately after immersion and returned to the baseline value 15 min after the immersion (compared with before: *p* < 0.01; *η*_*p*_^*2*^ > 0.26). HR increased from before to after immersion only in HWI-45°C (*p* < 0.001; *η*_*p*_^*2*^ = 0.67), and the time × trial interaction was significant (*p* < 0.001; *η*_*p*_^*2*^ = 0.36). The PSI was significantly greater in HWI-45°C than in WI-37°C (*p* < 0.001; *η*_*p*_^*2*^ = 0.63) (Figure 2E).

### Spinal and supraspinal reflex excitability

**FIGURE 3:**
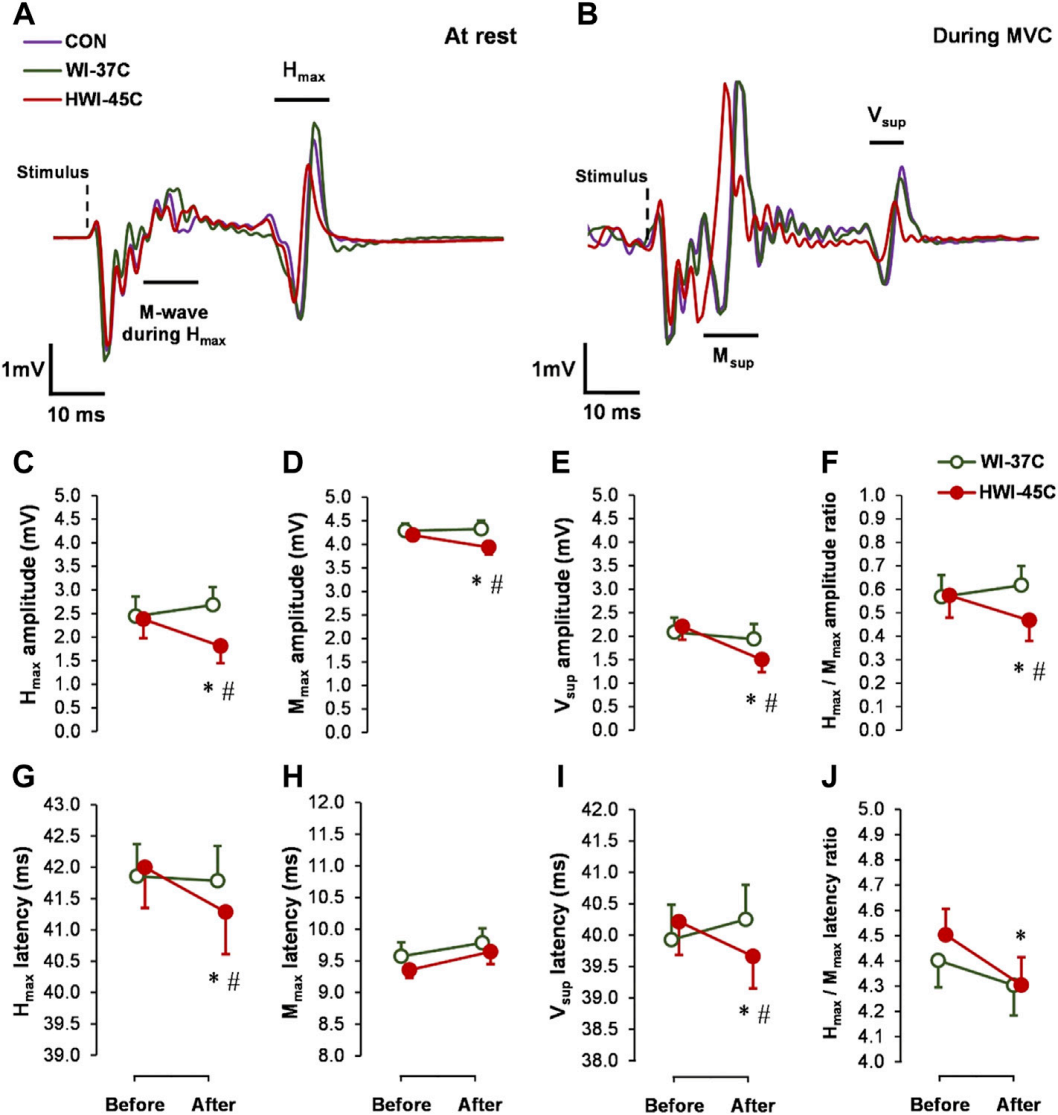
Examples of electrically induced waves recorded at rest **(A)** and during 5-s maximal voluntary contraction (MVC) **(B)** obtained in the control (CON) condition, and after whole-body immersion in water temperatures of 37 °C (WI-37°C) and 45°C (HWI-45°C). Changes in reflex amplitudes of H_max_ **(C)**, M_max_ **(D)**, and V_sup_ **(E)**, and the H_max_/M_max_ amplitude ratio **(F)**, and changes in the reflex latency of H_max_ **(G)**, M_max_ **(H)**, and V_sup_ **(I)**, and the H_max_/M_max_ latency ratio **(J)** of the soleus muscle of the dominant (right) leg from before to after WI-37°C and HWI-45°C. *p < 0.05, HWI-45°C compared with before; #*p* < 0.05, HWI-45°C compared with WI-37°C. Values are expressed as mean and SD.

The baseline H_max_ (Figures 3C,G), M_max_ (Figures 3D,H), and V_sup_ (Figures 3E,I) amplitude and latency did not differ between conditions (*p* > 0.05; *η*_*p*_^*2*^ < 0.05). Spinal and supraspinal reflex excitability did not change from before to after WI-37°C (compared with before: *p* > 0.05; *η*_*p*_^*2*^ < 0.09). H_max_ and V_sup_ amplitude and latency, as well as the H_max_/M_max_ amplitude and latency ratios decreased significantly in HWI-45°C (time effect: *p* < 0.05; *η*_*p*_^*2*^ > 0.22) (Figures 3F,J). These results suggest that spinal and/or supraspinal excitability rather than muscle sarcolemmal excitability were modulated. The H_max_ and V_sup_ amplitude and latency values, and H_max_/M_max_ amplitude ratio values were significantly lower after HWI-45°C than WI-37°C (trial effect: *p* < 0.05; *η*_*p*_^*2*^ > 0.25). A significant time × trial interaction was found (*p* < 0.05; *η*_*p*_^*2*^ > 0.31) for all effects. Resting M_max_ decreased only in response to HWI-45°C (time effect: *p* = 0.042; *η*_*p*_^*2*^ = 0.17), and the time × trial interaction was significant (*p* = 0.048; *η*_*p*_^*2*^ = 0.16).

### MVC, CAR, and SOL muscle sEMG

**FIGURE 4:**
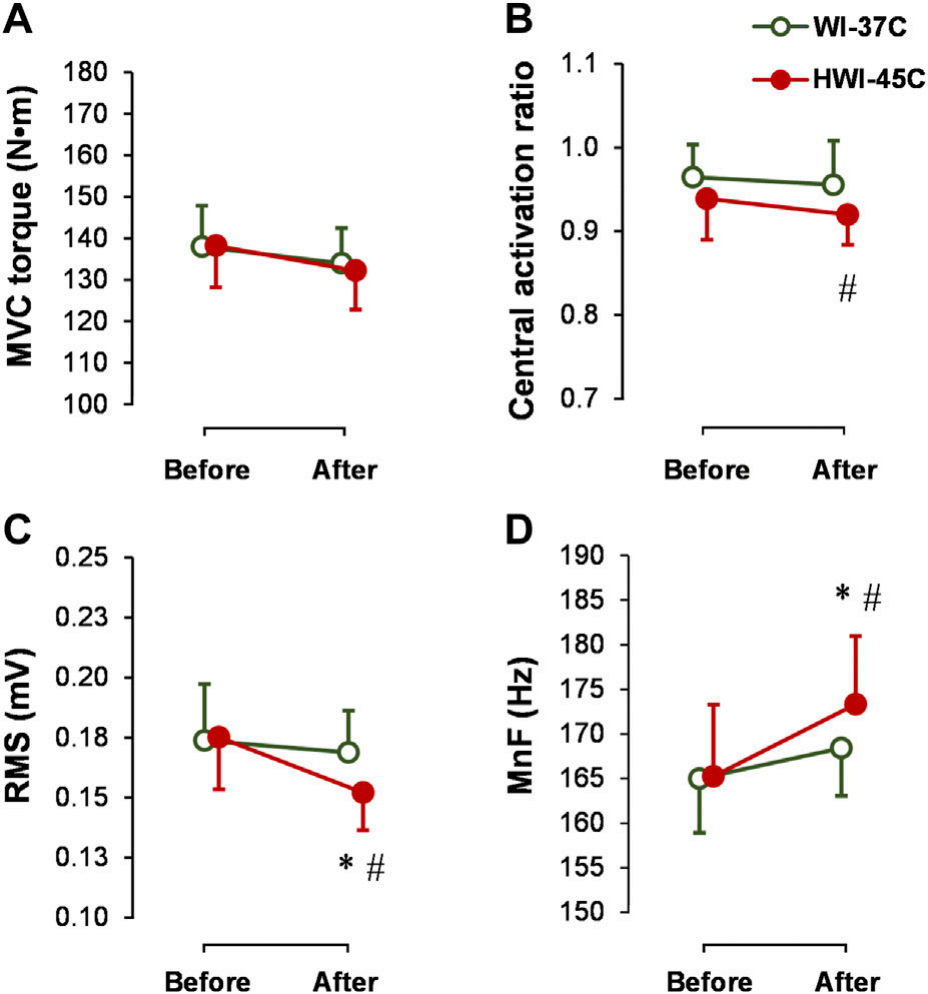
Changes in maximal voluntary contraction torque (MVC) **(A)**, central activation ratio **(B)**, and root mean square (RMS) **(C)**, and mean frequency (MnF) **(D)** of the sEMG signal of the soleus muscle during the 5-s MVC before and after whole-body immersion in water temperatures of 37°C (WI-37°C) and 45°C (HWI-45°C). *p < 0.05, HWI-45°C compared with before; #*p* < 0.05, HWI-45°C compared with WI-37°C. Values are expressed as mean and SD.

The baseline MVC torque (Figure 4A), CAR (Figure 4B), SOL-RMS (Figure 4C), and SOL-MnF of sEMG (Figure 4D) did not differ between conditions (*p* > 0.05; *η*_*p*_^*2*^ < 0.05). MVC torque and CAR did not change from before to after WI-37°C and HWI-45°C (compared with before: *p* > 0.05; *η*_*p*_^*2*^ < 0.08). CAR was lower after HWI-45°C than after WI-37°C (trial effect: *p* = 0.046; *η*_*p*_^*2*^ = 0.19), but the time × trial interaction was not significant (*p* = 0.582; *η*_*p*_^*2*^ = 0.06). SOL-RMS and SOL-MnF did not change from before to after WI-37°C (compared with before: *p* > 0.05; *η*_*p*_^*2*^ < 0.05). However, in parallel with the changes in the amplitude and latency of reflex H_max_ and V_sup_ excitability, SOL-RMS decreased and SOL-MnF increased from before to after HWI-45°C, and the time × trial interaction was significant (*p* < 0.05; *η*_*p*_^*2*^ > 0.25).

### Torque-generating capacity

**FIGURE 5:**
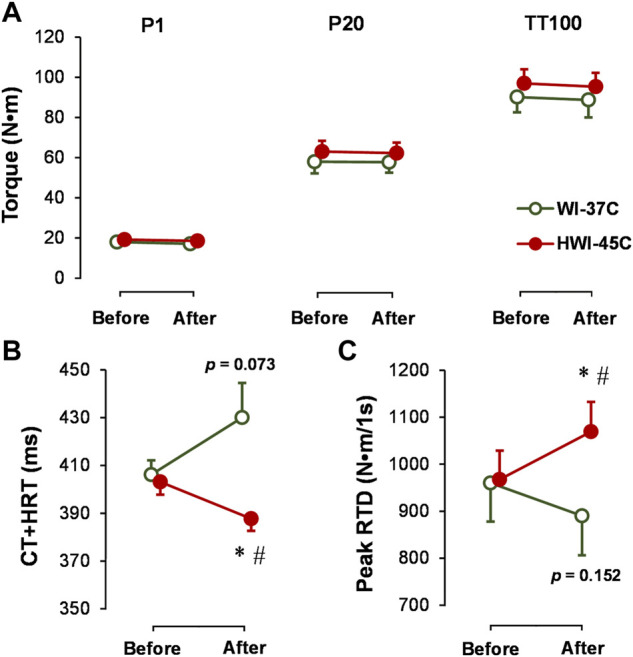
Changes in electrically induced ankle plantar flexion muscle isometric peak torque at 1 Hz (P1), 20 Hz (P20), and 25 stimuli at 100 Hz (TT100) **(A)**, and TT100-induced muscle contraction plus half-relaxation time (CT + HRT) **(B)** and peak rate of torque development (RTD) **(C)** before and after whole-body immersion in water temperatures of 37°C (WI-37°C) and 45°C (HWI-45°C). *p < 0.05, HWI-45°C compared with before; #*p* < 0.05, HWI-45°C compared with WI-37°C. The *p* values relate to the time effect in WI-37°C. Values are expressed as mean and SD.

The baseline electrically induced (P1, P20, and TT100) muscle peak torque production (Figure 5A), TT100-induced CT + HRT (Figure 5B), and peak RTD (Figure 5C) did not differ significantly between conditions (*p* > 0.05; *η*_*p*_^*2*^ < 0.05). Neither WI-37°C nor HWI-45°C was sufficient to alter the electrically (involuntary) induced peak torque (*p* > 0.05; *η*_*p*_^*2*^ < 0.04). Although TT100 peak torque was unaffected by both immersions, CT + HRT decreased (time × trial interaction effect: *p* = 0.0284; *η*_*p*_^*2*^ = 0.24), and peak RTD increased (time × trial interaction effect: *p* = 0.0198; *η*_*p*_^*2*^ = 0.26) after HWI-45°C. In contrast to HWI-45°C, in WI-37°C, CT + HRT tended to increase (time effect: *p* = 0.073; *η*_*p*_^*2*^ = 0.14) and peak RTD tended to decrease (time effect: *p* = 0.153; *η*_*p*_^*2*^ = 0.12).

### Central and peripheral fatigue during the 2-min MVC

**FIGURE 6:**
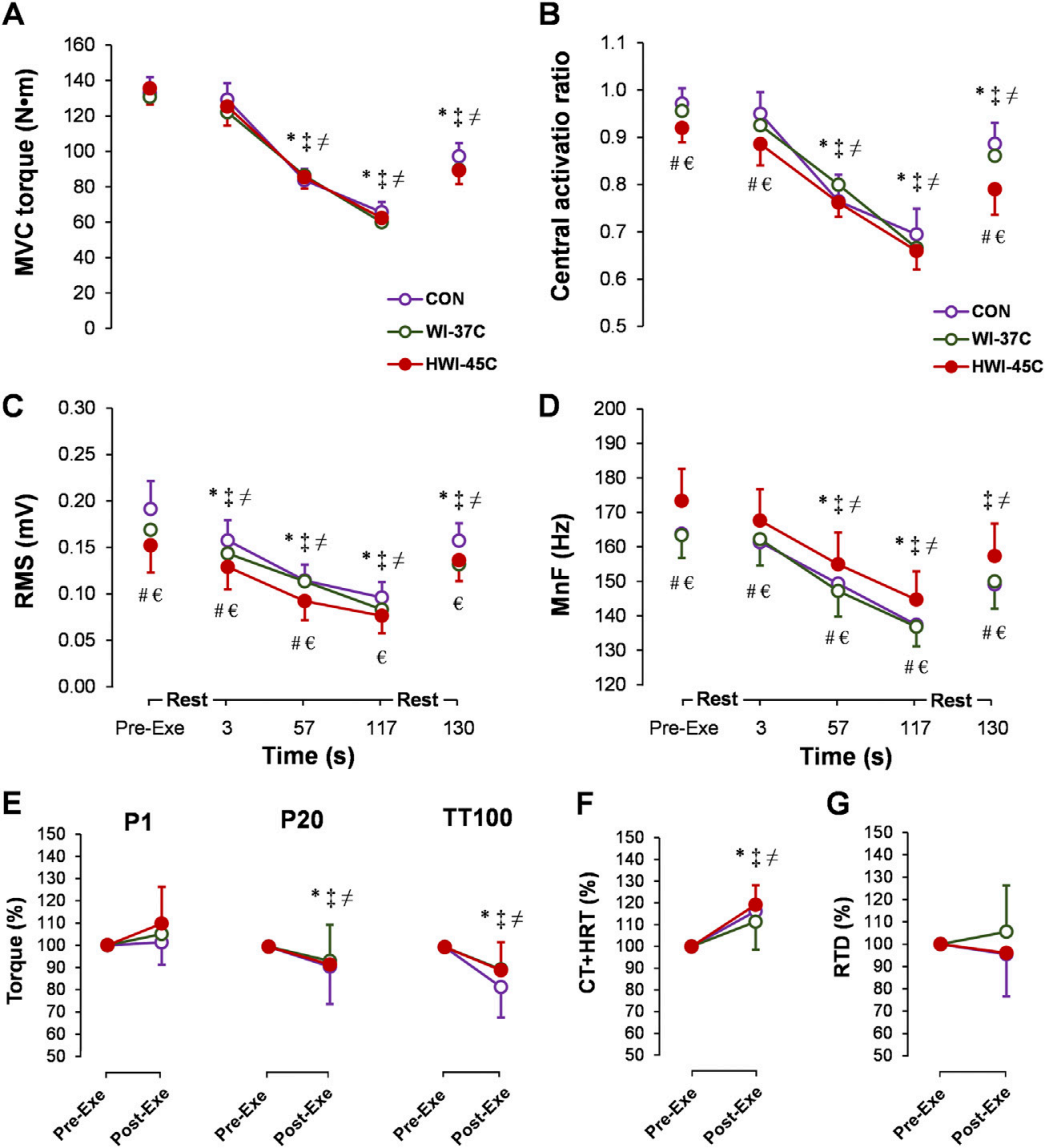
Time-dependent changes in maximal voluntary contraction torque (MVC) **(A)**, central activation ratio **(B)**, root mean square (RMS) **(C)**, and mean frequency (MnF) **(D)** of the sEMG signal of the soleus muscle during the 2-min MVC in the control (CON) trial and with whole-body immersions in water temperatures of 37°C (WI-37°C) and 45°C (HWI-45°C). Percentage changes from before to after the 2-min MVC in electrically induced torque **(E)**, TT100-induced muscle contraction plus half-relaxation time (CT + HRT) **(F)**, and peak rate of torque development (RTD) **(G)** in CON, WI-37°C, and HWI-45°C. *p < 0.05, HWI-45°C compared with before; ‡*p* < 0.05, WI-37°C compared with before; ≠ *p* < 0.05 CON, compared with before; #*p* < 0.05, HWI-45°C, compared with WI-37°C; € *p* < 0.05, HWI-45°C compared with CON. Values are expressed as mean and SD.

MVC torque decreased gradually during the exercise (main time effect: *p* < 0.001; *η*_*p*_^*2*^ = 0.64) (Figure 6A) but did not differ between conditions. CAR also decreased gradually during the exercise (main time effect: *p* < 0.001; *η*_*p*_^*2*^ = 0.49) and was lower before, at 3 s, and after exercise in HWI-45°C compared with the same times in CON and WI-37°C (Figure 6B) (main trial effect: *p* < 0.05; *η*_*p*_^*2*^ > 0.2). SOL-RMS (Figure 6C) and SOL-MnF (Figure 6D) decreased gradually during the exercise (main time effect: *p* < 0.001; *η*_*p*_^*2*^ > 0.5). In HWI-45°C, SOL-RMS was lower and SOL-MnF was higher during the 2-min MVC than in the two other conditions (main trial effect: *p* < 0.001; *η*_*p*_^*2*^ > 0.5). In all conditions, MVC torque, CAR, SOL-RMS, and SOL-MnF did not return to the preexercise value following the 10-s recovery (compared with preexercise: *p* < 0.01; *η*_*p*_^*2*^ > 0.35). P20 and TT100 peak torque decreased (Figure 6E) and TT100-induced CT + HRT increased (Figure 6F) from before to after the 2-min MVC (main time effect: *p* < 0.05; *η*_*p*_^*2*^ > 0.26). For all effects, no significant time × trial interaction was found (*p* < 0.05; *η*_*p*_^*2*^ > 0.31).

## Discussion

The present study investigated for the first time neuromuscular modulation in response to short-duration (5-min) exposure to noxious heat by immersion in water at 45°C. Short-duration whole-body immersion in 45°C water produced significantly greater thermophysiological strain (greater increases in T_re_, T_sk_, T_mu_, HR, and PSI) than immersion in innocuous warm water at 37°C. Some of these thermal effects (e.g., higher T_re_ and T_sk_) in HWI-45°C also persisted for 30 min after the end of immersion. The unique findings of our study are that short-duration immersion in noxious hot water decreased the amplitude and latency of spinal (H_max_) and supraspinal (V_sup_) reflexes, and that these changes were accompanied by decreased SOL-RMS and increased SOL-MnF. In parallel to the electrophysiological modulations, short-duration HWI-45°C decreased electrically TT100-induced CT + HRT and increased electrically TT100-induced peak RTD but did not change P1, P20, and TT100 peak torque values. Interestingly, these electrophysiological modulations in motor drive transmission as well as peripheral modulations in muscle contractility properties in response to HWI-45°C compared with the other two conditions seemed to have little effect on the central activation of exercising muscles (Figure 4B) and no effect on MVC torque production (Figure 4A) during brief MVC. Furthermore, no additional effects of provoked fatigue level on neuromuscular parameters were found during prolonged (2-min MVC) contraction.

To our knowledge, only one previous study has investigated the effects of acute short-duration (5-min) whole-body immersion in innocuous warm 37°C water on thermoregulatory and neurophysiological functions in normothermic compared with mildly hypothermic men (Brazaitis et al., 2016). That study reported a thermosensory excitation shift in response to 37°C water immersion through inhibition of body heat production in the mildly hypothermic men. In the same study, brief rewarming in warm water blunted the hypothermia-induced alterations in the transmission of neural drive and muscle contractility, despite lower T_re_ and deep T_mu_. During sustained 2-min MVC, additional failures at the supraspinal level seemed to impair motor drive to a greater extent in the briefly rewarmed, mildly hypothermic participants compared with the non-rewarmed condition (Brazaitis et al., 2016). In agreement with that previous study, here we found no effect on neurophysiological functions and performance after short-duration immersion in innocuous warm 37°C water in normothermic men. The appearance of thermoregulatory and neurophysiological alterations in response to short-duration (5-min) HWI-45°C and in response to short-duration rewarming of mildly hypothermic men in 37°C water (Brazaitis et al., 2016) suggests a shift in the activation threshold of the superficial thermo-TRP channels, which are most likely modulated by the initial T_sk_ (Tritsch, 1990).

In the present study, we found that the 5-min whole-body HWI in noxious 45°C water accelerated the contractile properties of calf muscles in normothermic men by increasing the speed of CT + HRT and the RTD peak while having no effect on electrically and voluntary induced peak torque production. These changes in the muscle contractile properties are linked to accelerated Ca^2+^ release and reuptake by the sarcoplasmic reticulum, and an increased rate of Ca^2+^ binding to troponin may account for faster activation of actin-myosin interactions in the HWI-45°C potentiated calf muscle (Fitts and Holloszy, 1978; Davies and Young, 1983; Segal et al., 1986). The tetanic peak force in SOL fibers is not altered when the temperature increases from 37 to 43°C, which suggests that muscle fibers can tolerate a temperature ∼6°C above the *in situ* temperature without displaying any sufficient change in tetanic force production (Place et al., 2009). However, changes in muscle contractility were accompanied by a decrease in resting M_max_ amplitude in our study, probably because of temperature-mediated effect on voltage-gated Na^+^ channels (Rutkove, 2001) and elevated ATP turnover (Gray et al., 2006). Specifically, the opening and closing of these channels accelerate at high temperature, and this allows less Na^+^ to enter cells and leads to a more rapid onset of depolarization (Rutkove et al., 1997).

The increase in peak contraction and relaxation speeds in the muscle means that higher rates of motoneuron discharge would be required to sustain a given level of torque (Segal et al., 1986). We found that, following HWI-45°C, SOL-RMS decreased and SOL-MnF increased despite no change in peak MVC torque production. These changes were accompanied by decreases in the amplitude and latency of H_max_ and V_sup_. However, it has been suggested that such neuromuscular modulation in response to increased whole-body temperature may directly affect centrally driven voluntary activation of exercising muscles (Todd et al., 2005). Intriguingly, in our study, the preexercise CAR was lower in HWI-45°C than in the other two conditions (Figure 6B), and brief MVC peak torque production was unchanged after immersion. These findings suggest that HWI-45°C potentiated muscle contractility (i.e., made it faster) and that lower centrally driven activation is required for a similar voluntary torque production. The impairment in activating heat-potentiated muscle centrally during brief and sustained MVC to the same extent as in the thermoneutral condition may reflect inadequate input to the motoneuron pool or the inability of motoneurons to respond to adequate input (Todd et al., 2005).

To provoke inhibition of the central drive to exercising muscles and to investigate whether noxious HWI-45°C can induce modulation of muscle contractility and whether spinal and supraspinal excitability can persist and contribute to lower resistance to central and/or peripheral fatigability, the participants in the present study performed a sustained 2-min MVC (Bernecke et al., 2015). We hypothesized that the increased neuromuscular tension required to produce fusion of torque in HWI-45°C would pose a greater energetic requirement during the sustained 2-min MVC (Rome and Kushmerick, 1983) by providing greater inhibitory feedback to central structures, which may contribute to the greater central fatigue (Bigland-Ritchie et al., 1986). In contrast to our expectation, even though HWI-45°C caused neuromuscular modulation, it did not seem to increase additional peripheral or central fatigue during the 2-min MVC. This contrasts with evidence that WBH decreases the neural drive transmission at both the level of the peripheral nervous system and spinal cord, and decreases the capacity to sustain voluntary activation of exercising muscles for more than a few seconds (Nybo and Nielsen, 2001; Todd et al., 2005; Racinais et al., 2008; Brazaitis et al., 2010; Périard et al., 2014).

Both MVC torque and central voluntary activation during 10-s MVC decrease gradually with an increase in core temperature (up to 39.5°C) (Morrison et al., 2004). *In vitro*, the stimulation of a nerve causes the release of a variable number of quanta per impulse during a train of stimuli; however, this is termed a failure if the postsynaptic quantal units are undetected following a stimulus (Karunanithi et al., 1999). In a study of *Drosophila* synapses by Karunanithi et al. (1999), at 22°C, all synapses produced one or more quantal events for each nerve impulse without any failure. However, the amplitude of the response declined as the temperature increased, and failure became evident until transmission failed completely when the nerve temperature reached 35°C. Together, these studies suggest that, in our study, the increase in core (∼0.2°C) and muscle (∼0.6°C) temperatures were far from the moderate/severe WBH needed to affect central structures and induce greater central fatigue during the 2-min MVC.

Temperature plays a central role in the effects of many thermal therapy modalities that are used for recovery and treatment, and to maintain physical and mental well-being. Our results suggest that a high body temperature (hyperthermia) rather than short-duration HWI-45°C potentiated (accelerated) neuromuscular energetics most likely reflects additional neuromuscular failure during fatigue-provoking exercise. However, the question remains whether acute superficial thermally induced neural stimulation can and should be used as an alternative warm-up method before an event to improve physical performance. As the superficial body area is heated acutely, the potentiated neural excitability associated with accelerated muscle contractility and greater torque development may be regarded as a way to increase physical performance in an athlete, similar to that seen after a physical warm-up, without leading to a higher risk of neuromuscular failure and fatigue if the core temperature increases to a critical level.

This study has some limitations. Generalizability of our findings may be limited, as only participants not meeting physical activity guidelines were recruited in this study, and our findings may therefore not be representative of the athletic and physically active population. Second, only males participated in this study, and it could not be determined if sex played a role.

In conclusion, we have shown that short-duration (5-min) whole-body immersion in noxious 45°C water produced a greater superficially induced heat transfer to the body core and deep muscles, and evoked a greater thermoregulatory response than immersion in innocuous warm 37°C water in normothermic men. Exposure to noxious heat accelerated muscle contractility but did not change electrically and voluntary induced peak torque production. Accelerated muscle contractility was accompanied by electrophysiological modulation/adjustment, including decreases in the amplitude and latency of spinal and supraspinal reflexes, and by a decrease in amplitude and increase in the frequency component of surface electromyography activity of the working SOL muscle. Interestingly, these neuromuscular modulations in response to short-duration 45°C water immersion seemed to have no additional effect on central and peripheral fatigue during the 2-min MVC.

## Data availability statements

The original contributions presented in the study are included in the article/Supplementary Material, further inquiries can be directed to the corresponding author.
